# Recent advances in treatment of follicular lymphoma: efficacy of PI3Kα/δ inhibitor (TQ-B3525)

**DOI:** 10.1038/s41392-024-01842-z

**Published:** 2024-05-16

**Authors:** Takuya Watanabe

**Affiliations:** Department of Internal Medicine and Gastroenterology, Watanabe Internal Medicine Aoyama Clinic, Niigata-city, 9502002 Japan

**Keywords:** Target validation, Drug development

The commented study reports the results of monotherapy using TQ-B3525, a novel phosphatidylinositol-3 kinase (PI3K) inhibitor developed in China, in patients with refractory/relapsed follicular lymphoma (r/r FL).^1^

## Recent PI3Ki-related advances

B-cell receptor (BCR) signaling pathway activation in B-cell tumors has led to the development of specific inhibitors targeting this cascade. PI3K is central to this pathway, being crucial for inositol phospholipid phosphorylation in the cell membrane. Among the diverse class I PI3Ks, the α, β, and δ isoforms are significant. Malfunction in these isoforms leads to various cancers, including B-cell malignancies.

Notably, PI3Kis have been developed, with idelalisib targeting the delta isoform, which yielded promising results. In the Caucasian population, idelalisib produced impressive response rates and progression-free survival (PFS) in r/r FL, highlighting its therapeutic potential in B-cell cancers. A U.S. phase II study demonstrated that idelalisib yielded a 57% response rate and an 11-month median PFS in patients with r/r indolent non-Hodgkin lymphoma (iNHL), including FL.^2^ Despite its high efficacy, another study focusing on rituximab combined with lenalidomide was stopped due to severe toxicity, especially in younger, previously untreated patients with a strong immune system.

Duvelisib, the first FDA-approved oral dual PI3Kδ/γ inhibitor, demonstrated a relatively high CR rate in patients with iNHL/FL, although its high incidence of serious adverse events and safety concerns remain unresolved.

Copanlisib, approved for FL after multiple therapy lines, displayed promising results in the CHRONOS-1 trial for r/r iNHL, with a 59% and 12% overall response rate (ORR) and complete response (CR), respectively, as well as median PFS and overall survival (OS) of 11 and 43 months, respectively.^3^ However, it induced severe (grade 3 or higher) side effects in 84% of the patients, including six fatal outcomes.^3^

Umbralisib, an orally administered, selective PI3Kδ and CK1ε inhibitor, emerges as a fourth-generation PI3Ki for cancer treatment. However, the observed ORR of 45% was not superior to that of other PI3K inhibitors.

Parsaclisib, a selective delta-isoform PI3Ki, yielded promising results in the CITADEL-111 phase Ib study, with 100% and 22.2% ORR and CR, respectively, in Japanese patients with r/r FL, although its adverse events necessitated cautious patient selection.^4^ A Parsaclisib-related phase II trial is currently ongoing.

Zandelisib (ME-401), a new PI3Kδ inhibitor, demonstrated high efficacy in Japanese r/r iNHL patients, with 100% and 22% ORR and CR, respectively. The median response duration, PFS, and time-to-response were 7.9, 11.1, and 1.9 months, respectively.^5^ In phase Ib trial for r/r FL, zandelisib monotherapy resulted in ORRs of 92% and 83% in the case of 60 and 180 mg, respectively. The phase III CHRONOS-4 trial indicated higher ORRs than those upon the administration of bendamustine with rituximab (BR) or rituximab-CHOP in patients with r/r iNHL. Severe adverse events affected 70% and 91% of the patients in the BR group and BR and R-CHOP groups, respectively.

Certain remaining comments related to the hereby-discussed study should also be addressed. First, although the clinical trial in this study has limitations in that (1) it was a single-arm and not a group comparison study and (2) it only included Chinese and not multiracial patients (e.g., White or Black individuals), considering that the target population of the clinical trial consisted of patients in advanced r/r FL stages comprised, conducting a group comparison with the placebo group trial would be particularly challenging. Therefore, this single trial is of immense clinical significance.

Duvelisib, copanlisib, and linperlisib have been recently approved in China. However, at the time of the commented study, no PI3Ki had been approved in China. The following PI3Kis were tested in r/r FL in the USA and Europe: idelalisib, duvelisib, and copanlisib. Only copanlisib remained in trials in the USA due to its severe side effects. Subsequently, the PI3K inhibitors umbralisib, parsaclisib, and zandelisib were developed. Combined therapies of copanlisib with BR/R-CHOP (CHRONOS-4trial) as well as idelalisib with lenalidomide were tested. Nonetheless, the trials were stopped due to the various adverse effects.

However, TQ-B3525, developed in China, has attracted worldwide attention due to its increased efficacy compared to idelalisib and copanlisib in this single-agent trial and the higher level of tolerance of adverse reactions. The authors also discuss the reasons why TQ-B3525 yielded better therapeutic efficacy and fewer adverse events than existing PI3Kis, which we find particularly interesting.

The study also points out that patients with r/r FL and more than three prior treatments might exhibit increased TQ-B3525 effectiveness, resulting in a higher therapeutic effect. The reasons for this are discussed by the authors.

TQ-B3525 should also be considered in combination with tazemetostat (EZH2 inhibitor) and mosunetuzumab (CD20 x CD3 bispecific antibody), which, until recently, were not approved in China. Combination therapy involving TQ-B3525, either alone or with other novel agents, is expected to further improve the outcomes of patients with FL.

**Fig. 1:**
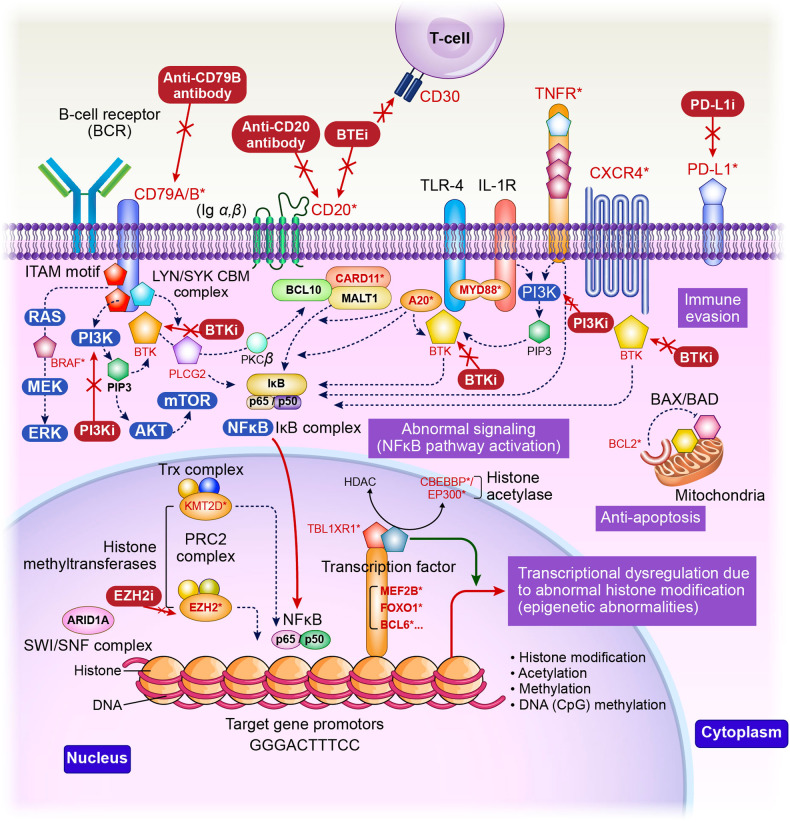
Follicular lymphoma (FL)-related genes involved in FL pathogenesis and proliferation, and the action of novel therapeutic agents targeting them

The recent development of novel FL therapeutics has been remarkable. The novel therapeutic agents include: (1) mAb/ADC (anti-CD20/CD79b); (2) Bispecific T cell binding antibody (CD20 × CD3 bispecific antibody); (3) Anti-programmed death ligand 1 (PD-L1) antibody; (4) Immunomodulatory drugs such as lenalidomide; (5) Molecular targeted therapies (small-molecule compounds), comprising Bruton’s tyrosine kinase inhibitor, B-cell lymphoma 2 (BCL2) inhibitor, Epigenetic regulators (EZH2), PI3Kis, and PI3K/AKT/mechanistic target of rapamycin (mTOR) pathway inhibitor; (6) Chimeric antigen receptor (CAR)-T cell therapy (Fig. 1).

Although various novel therapeutic agents have been discovered and yielded promising therapeutic results, how these novel agents should be combined and the application order producing the most effective results remains elusive. With various new therapeutic agents being recently developed in such a short period, FL treatment strategy-related decisions might appear to be somewhat chaotic. Nevertheless, all these advances could bring us closer to the hopeful curability of FL in the near future.
